# The Effectiveness of Paired Associative Stimulation on Motor Recovery after Stroke: A Scoping Review

**DOI:** 10.3390/neurolint16030043

**Published:** 2024-05-14

**Authors:** Andrea Baroni, Annibale Antonioni, Giulia Fregna, Nicola Lamberti, Fabio Manfredini, Giacomo Koch, Alessandro D’Ausilio, Sofia Straudi

**Affiliations:** 1 Department of Neuroscience and Rehabilitation, University of Ferrara, 44121 Ferrara, Italy; brnndr3@unife.it (A.B.); giulia.fregna@unife.it (G.F.); nicola.lamberti@unife.it (N.L.); fabio.manfredini@unife.it (F.M.); giacomo.koch@unife.it (G.K.); alessandro.dausilio@unife.it (A.D.); strsfo@unife.it (S.S.); 2 Department of Neuroscience, Ferrara University Hospital, 44124 Ferrara, Italy; 3 Doctoral Program in Translational Neurosciences and Neurotechnologies, University of Ferrara, 44121 Ferrara, Italy; 4 Center for Translational Neurophysiology of Speech and Communication (CTNSC), Italian Institute of Technology (IIT), 44121 Ferrara, Italy; 5 Non Invasive Brain Stimulation Unit, Istituto di Ricovero e Cura a Carattere Scientifico Santa Lucia, 00179 Rome, Italy

**Keywords:** paired associative stimulation (PAS), stroke, neurorehabilitation, non-invasive brain stimulation (NIBS), plasticity, neurophysiology

## Abstract

Paired associative stimulation (PAS) is a non-invasive brain stimulation technique combining transcranial magnetic stimulation and peripheral nerve stimulation. PAS allows connections between cortical areas and peripheral nerves (C/P PAS) or between cortical regions (C/C PAS) to be strengthened or weakened by spike-timing-dependent neural plasticity mechanisms. Since PAS modulates both neurophysiological features and motor performance, there is growing interest in its application in neurorehabilitation. We aimed to synthesize evidence on the motor rehabilitation role of PAS in stroke patients. We performed a literature search following the PRISMA Extension for Scoping Reviews Framework. Eight studies were included: one investigated C/C PAS between the cerebellum and the affected primary motor area (M1), seven applied C/P PAS over the lesional, contralesional, or both M1. Seven studies evaluated the outcome on upper limb and one on lower limb motor recovery. Although several studies omit crucial methodological details, PAS highlighted effects mainly on corticospinal excitability, and, more rarely, an improvement in motor performance. However, most studies failed to prove a correlation between neurophysiological changes and motor improvement. Although current studies seem to suggest a role of PAS in post-stroke rehabilitation, their heterogeneity and limited number do not yet allow definitive conclusions to be drawn.

## 1. Introduction

Physiological reactions are frequent following stroke and aim to repair the damaged tissue. Plasticity refers to the ability of the brain to modify its structure and function in response to experience and environmental demand [1]. This enhanced plasticity following brain damage leads to new axon sprouting, new synapse formation, and the remapping of sensory–motor areas [2]. Several studies confirm a close relationship between neuroplasticity and functional recovery following stroke [3]. Changes in the activity and connection between neurons can be identified around the lesion up to remote areas or in the contralateral hemisphere, explaining spontaneous recovery after cerebral damage [4]. Post-stroke rehabilitation aims to improve functional recovery and promote neuroplasticity, supporting this dynamic process in rebuilding connections between neurons [5]. Non-invasive brain stimulation (NIBS) techniques are a promising adjuvant strategy for enhancing post-stroke recovery through the modulation of cortical excitability and neuronal plasticity [6]. The combination of NIBS and motor or behavioral intervention has gained substantial interest over the last years due to the promising potentiality that the combined approach offers [3]. Several studies on post-stroke patients combined NIBS and rehabilitative approaches such as intensive physiotherapy or occupational therapy [7,8], robot-assisted training [9,10,11], virtual reality rehabilitation [12,13,14,15], and task-oriented training [16] for promoting motor recovery. Between the available NIBS techniques, transcranial magnetic stimulation (TMS) has been used to investigate and induce plasticity in the human brain [17]. The combination of TMS and peripheral nerve electrical stimulation (PNS) is known as paired associative stimulation (PAS). PAS is an emerging NIBS approach, introduced by Stefan et al. [18], which uses the Cell Assembly Theory first formulated by Hebb in 1949 [19,20]. Hebb postulated that repeated activation of a presynaptic cell immediately before the activation of a postsynaptic cell induces synaptic strengthening, so-called long-term potentiation (LTP). Hebb did not propose an opposite activity-dependent reduction in synaptic strength or long-term depression (LTD). Indeed, later work described a heterosynaptic LTD, when a presynaptic cell repeatedly and persistently fails to excite the postsynaptic cell [21], and a homosynaptic mechanism based on low-frequency stimulation of the presynaptic element [22]. Studies on animal models have shown how PAS can influence motor cortex excitability, whereas TMS or PNS, commonly used in rehabilitation, showed no significant effect when used alone [23]. PAS’s effect on the human brain was first studied on healthy subjects, and the observed increase in Motor Evoked Potential (MEP), the response induced by a TMS pulse over the Primary Motor Cortex (M1), suggested the plasticity of brain structures [24,25]. Many single-session studies explored the effects of different PAS protocols in stroke patients [26,27,28]. Even though promising results were found on cortical excitability and motor performance, no results were found on repeated sessions of PAS, particularly when combined with rehabilitative treatment. Several mechanisms may justify the use of PAS-empowered rehabilitative approaches: First, the increase in corticomuscular excitability induced by PAS may favor the subsequent response to neurorehabilitation treatment [28,29]. Furthermore, PAS protocols act on circuits involved in use-dependent plasticity, reinforcing connections useful for performing a specific motor task during rehabilitation [30,31]. However, although the effectiveness of PAS in stroke rehabilitation is still unclear, the emerging interest in this NIBS technique makes it necessary to summarize the current evidence on PAS-empowered motor rehabilitation. Thus, this work aims to review the available literature on PAS for motor rehabilitation following stroke. Moreover, we aimed to provide information about parameters and sites of stimulation, as well as outcomes and patients who could benefit from PAS. Due to the heterogeneity of evidence in this field, we applied a scoping review approach following the Preferred Reporting Systems for Systematic Reviews and Meta-Analyses Extension for Scoping Reviews (PRISMA-ScR) Framework [32].

## 2. Materials and Methods

The protocol of this scoping review has been redacted following the PRISMA-ScR Framework and has been pre-registered on an Open Science Framework (OSF) with the following doi: https://doi.org/10.17605/OSF.IO/86UAC (accessed on 20 October 2022). 

### 2.1. Search Strategy

The PICO framework was used to define the research question. Articles published in peer-reviewed journals and pre-peer-reviewed web publications were potentially eligible for inclusion. The literature search was performed in the following electronic bibliographic databases: PubMed, Web of Science, Science Direct, and Embase. The database search was completed on 21 September 2022 and frequently updated until 31 December 2023. The search strategy included a controlled vocabulary and keywords adapted to the characteristics of the single database. A comprehensive description of the search strategy is available as a Supplemental Material (see Supplementary Materials). All the studies carried out on post-stroke adult patients where a PAS treatment was applied for the rehabilitation of motor function were considered. Only studies applying more than a single session on consecutive days were included. No restrictions on rehabilitation settings were used. 

### 2.2. Study Inclusion/Exclusion Criteria

The study population includes adult stroke patients, without regard to the type of lesion (ischemic or hemorrhagic), time from injury, and site of brain damage, who underwent PAS as a rehabilitation treatment, in combination or not with other rehabilitation techniques. We considered eligible multi-session clinical trials (RCT, nRCT, and pre–post studies) with or without a comparator. Inclusion criteria were (i) reference in English; (ii) study subjects and setting as described above; and (iii) studies that describe the application of PAS as a rehabilitative approach for upper or lower limb in stroke patients. Exclusion criteria were (i) studies regarding PAS in patients with different pathologies other than stroke; (ii) studies evaluating the effects of PAS on non-motor outcomes in stroke patients (i.e., dysphagia).

### 2.3. Study Selection

**Figure 1:**
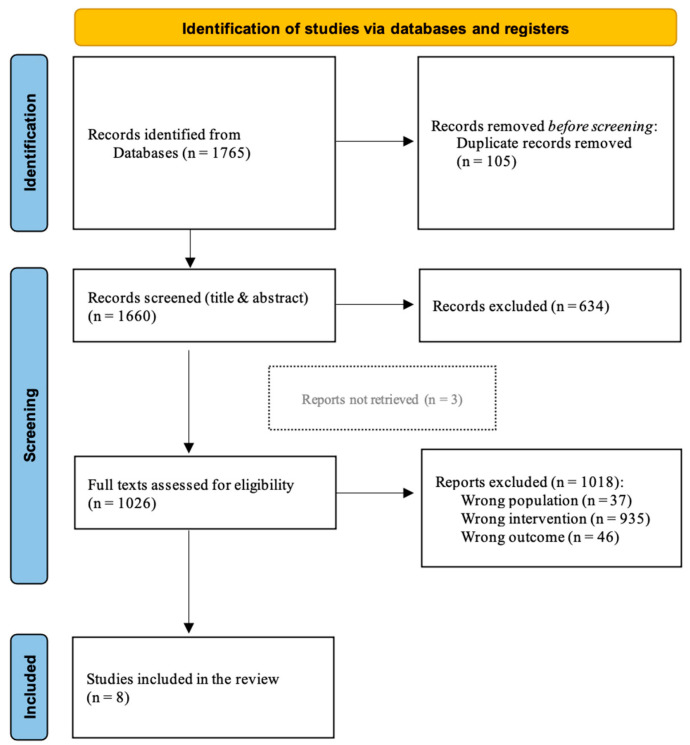
PRISMA review flow-chart.

Duplicate articles were excluded. Two independent reviewers (A.A. and G.F.) screened the title and abstract, and disagreement between them was solved by a third reviewer (A.B.). A.A. and G.F. reviewed the full text of the selected studies, and discordance was solved by A.B. and/or S.S. (see Figure 1).

### 2.4. Data Extraction

Two authors (A.B. and A.A.) independently extracted data using a pre-defined framework. The data framework included a field for the author(s), year of publication, country of origin, study design, sample size, type of stroke, time from stroke, PAS parameters (type, points of application, intensity and frequency of stimulation, ISI, and time of application), associated treatments, comparator details, outcome measures, and possible adverse effects related to treatment. The critical appraisal of the included papers was performed using the Cochrane Risk of Bias Tool (RoB) for RCTs [33]; nRCT and pre–post studies were evaluated using the Joanna Briggs Institute (JBI) critical appraisal tool [34]. Due to the nature of the project and the heterogeneity of the included studies, a narrative collection of results was planned.

## 3. Results

Database searching identified 1765 records. After removing duplicates, 1660 records were screened for the title and abstract and 634 records were excluded. Of the 1026 remaining papers, 1018 did not meet inclusion criteria and were excluded from the collection: 37 studies applied PAS to a different population, 935 studies applied a different stimulation protocol, and 46 studies evaluated a different outcome. Among the eight remaining studies [35,36,37,38,39,40,41,42], three full texts were unavailable [39,40,41]; however, we decided to include them in the scoping review due to the poor literature on the topic (Figure 1). Seven included studies were RCTs [34,35,36,37,38,39,40], and only one was a case series study [42]. All the studies were published in the last 20 years with a wide geographic distribution: four in France and one each in China, Turkey, Australia, and Ukraine. Most of the studies involved patients with ischemic stroke [35,36,38,39], two studies involved stroke patients without distinction between hemorrhagic or ischemic etiology [37,42], and two studies did not specify stroke origin [40,41]. Two studies involved chronic stroke patients (>six months post-stroke) [35,42], five studies involved subacute patients (1 to 6 months post-stroke) [36,37,38,40,41], and one study did not specify the time from stroke onset [39]. A total of 288 subjects were recruited. The median number of patients involved in the studies was 27.5 patients (IQR 24.75–40.25); of them, 16 (IQR 13.5–20.0) were male (one study did not specify this data [34]). Using available data, a median number of 13 (IQR 11.5–15) patients received real stimulation with different PAS protocols (one study did not specify patients’ distribution among treatment groups [39]). A detailed description of the included studies is reported in Table 1 and Table 2. 

### 3.1. PAS Procedures

C/P stimulation was the PAS type most used in the included studies [36,37,38,39,40,41,42]. Only one study adopted a C/C protocol [35]. Most studies used the PAS protocol for upper limb treatment [35,36,37,38,39,40,41]. Only one of the C/P studies aimed to improve lower limb function and gait [42].

#### 3.1.1. Cortico-Peripheral PAS

TMS was applied over the lesioned M1 in two of the included C/P studies [37,40]. One study stimulated the contralesional M1 [38]. One study stimulated both lesional and contralesional M1 in two different groups of treatment [36]. Three studies did not specify the TMS point of application [39,41,42]. Only two studies specified TMS intensity and stimulation frequency [36,38]. PNS was applied to the affected upper [37,41] or lower [42] extremity in three cases. One study used PNS in both hands in two different groups of treatment [36]. Two studies specified the site of stimulation but not the side [38,40]. One study did not specify the PNS point of application [39]. Only four studies defined both these parameters regarding the intensity and frequency of PNS [36,37,38,42]. Only four studies specified the ISI [36,37,40,42]: three of them applied the two stimuli with an ISI of 25 ms or 35 ms for an LTP effect [37,40,42]; and one used an ISI of 25 ms or 10 ms in two different groups of treatment to achieve LTP or LTD, respectively [36].

#### 3.1.2. Cortico-Cortical PAS

The study that applied C/C PAS stimulated the contralesional cerebellum and the lesional M1, specifying intensity, the frequency of stimulation, and ISI [35].

### 3.2. Treatment Duration

A median number of 10 (IQR 5–19.5) sessions of PAS was applied in the included study, with a minimum of 5 [35,37,40,41] and a maximum of 28 [36,42]. Only two studies quantified PAS duration in 30 min [41,42].

### 3.3. Associated Treatments

Four studies combined PAS with motor rehabilitation [35,36,37,38]. Specifically, three studies applied PAS before rehabilitation treatment [35,36,38], whereas one did not specify the order of the combined treatment [37]. The motor rehabilitation consisted of active-assisted range of motion exercises combined with motor imagery, strength training against gravity, and task-specific training [35]; good limb placement, bed movement, transfer training, operation treatment, daily life activity training, and other comprehensive rehabilitation treatment [36]; and activities to improve strength, flexibility, transfers, posture, balance, coordination, and activities of daily living [38]. Four studies did not specify these data [39,40,41,42]. 

### 3.4. Comparators

The most used comparator treatment was sham stimulation [35,37,39,40]: two studies delivered it through a sham coil applied following the same procedures used for real stimulation [35,37]; and two studies applied sham stimulation without describing the sham procedure [39,40]. One study applied a not-specified placebo as a treatment comparator [41]. Two studies used only physical therapy for the patients assigned to the control group [36,38].

### 3.5. Outcome Measures

#### 3.5.1. Neurophysiological Measures

MEP, Resting Motor Threshold (RMT), and functional Magnetic Resonance Imaging (fMRI) were used to assess the effects of PAS on corticospinal excitability. MEP was the most used outcome measure of PAS efficacy [35,36,37,39,40,41,42]. Six studies reported an increase in MEP amplitude [35,36,39,42] and/or surface area (the level of corticospinal projections excitability of the target muscle) [37,39,41] in the experimental group compared to the control; however, out of all these, only four studies reported quantitative results [35,37,41,42] but no statistically significative results in both within- and between-group comparisons. One study reported a significant increase in the MEP amplitude of groups who received PAS in different protocols without reporting quantitative data (only graphs available) [36]. RMT is the amount of TMS machine output necessary to produce an MEP that exceeds an established peak-to-peak amplitude (usually 50 μV) 50% of the time in a finite number of trials [43,44]. RMT was recorded in two studies [36,39]: one study reported a significant reduction in RMT of the lesioned side in the groups who received real stimulation, only through qualitative and graphical results [36]. Kuznietsova et al. described a reduction in RMT in the experimental group without reporting numerical data [39]. fMRI was recorded in two studies and revealed increased activation of the affected hemisphere without reaching statistical significance [35,38].

#### 3.5.2. Clinical Measures

The efficacy of PAS on upper limb function was evaluated using the Fugl-Meyer Assessment for Upper Extremity (FMA-UE) [35,36,37,39,40], the Motricity Index (Upper Extremity section) (MI-UE) [38], and the upper extremity section of the Brunnstrom Recovery Stages (BRS-UE) [38]. One study reported a significant improvement in FMA-UE score in the experimental group compared to the control, reporting only qualitative data and graphical results [36]. All other studies evaluating upper limb function did not record significant differences between the experimental and the control group [37,38,40,41]. Changes in hand function following PAS were evaluated using the Jebsen Hand Function Test (JHFT) [35], the grip strength (GS) [35], the Simple Test for Evaluating Hand Function (STEF) [36], and the hand section of the Brunnstrom Recovery Stages (BRS-H) [38]. No significant changes were recorded between the study groups. No effects of PAS on muscle tone [38], cognitive, and emotional function [36] were documented. Two studies assessed the efficacy of PAS on the ability to perform activities of daily living [36,38]. Of these, one study reported a significant improvement in the Barthel Index score only in the group that received real stimulation [36]. The efficacy of PAS on lower limb function (maximum voluntary contraction and range of motion) and activities (walking) was evaluated, and no changes were found between pre- and post-treatment [42]. Table 3 summarizes these data.

### 3.6. Adverse Effects

Possible adverse effects of PAS were recorded only in three of the included studies [35,37,38]. Two subjects showed temporary headaches after stimulation: both received C/C PAS and were allocated one in the real stimulation group and one in the control group [35]. One subject showed reflex syncope immediately after the end of the C/C sham PAS [35]. Two studies did not report adverse effects [37,38].

### 3.7. Quality Assessment

Considering the risk of bias evaluation of the included studies for which the full text was available [34,35,36,37,41], a heterogeneous methodological quality was noticed (Table 1). Particularly, among the RCTs involved, two studies showed an overall low risk of bias [35,38], while in the other two [36,37], the absence of explicit information on different methodological key points did not allow a precise estimation of the related methodological quality [32].

## 4. Discussion

Although recent studies have provided a better understanding of the neurophysiological mechanism underlying PAS, and supporting its contribution to stroke recovery, few studies have specifically investigated its role in rehabilitation. This lack may be due to their relatively recent introduction in the clinical setting, which makes further studies necessary to evaluate the applicability of this technique for patient recovery [45]. Moreover, the technologies required to implement PAS into practice are extremely expensive and require specific skills not always available outside the research settings [46]. However, considering their potential from a neurorehabilitation perspective, here, to the best of our knowledge, we have gathered evidence on PAS-empowered post-stroke motor rehabilitation. In our review, we aimed to synthesize the state-of-the-art of PAS as an adjuvant to stroke rehabilitation, identifying parameters, sites of stimulation, and patients who can benefit from this combined stimulation. The implications of our results will be discussed, considering the C/P and C/C PAS studies separately.

### 4.1. C/P PAS

M1 represents the most frequently stimulated area due to its relatively easy accessibility with NIBS techniques, as well as the possibility of measuring the effects of its modulation (e.g., RMT and MEPs recorded from target muscles) [43,44]. Moreover, M1 is a crucial part of a wide network responsible for the regulation of motor acts where sensory stimuli, exogenous and endogenous, play a key role [47].

Several studies have shown that PNS can inhibit the subsequent homotopic muscle response evoked by a TMS pulse on M1, leading to a decrease in MEP amplitude, depending on the specific temporal interval between the sensory and the motor stimulus [48]. This phenomenon is referred to as short-latency afferent inhibition and highlights a close coupling between sensory and motor networks, dependent on the modulation of inhibitory circuits exerted by excitatory cholinergic thalamocortical afferents [48]. Therefore, considering the importance of sensorimotor integration in motor control, it is unsurprising that PAS protocols target M1 in combination with accessible peripheral regions [28,49]. Consistently, most of the included studies stimulated the impaired hand, with a particular focus on the extensor muscles, frequently impaired after stroke. By contrast, few studies stimulated the median or, generically, the whole paretic hand [50]. Interestingly, only one study applied PAS stimulation to both hands, using an excitatory protocol on the paretic one and an inhibitory protocol on the healthy one [36]. This study design is present (when the information is available) in most of the other included studies. However, limited to the paretic hand, it is based on the model developed by Di Pino et al. on post-stroke interhemispheric disequilibrium: after stroke, the normal reciprocal inhibition between the two hemispheres is altered and the damaged hemisphere is no longer able to adequately counteract the healthy one, which therefore exerts a marked inhibition on the injured hemisphere hindering the recovery of impaired functions [51]. Although recent models have considered the role of other factors in addition to the mere distinction between the injured and healthy hemisphere, this interpretation has been widely used showing remarkable efficacy in the recovery of common symptoms after stroke [52,53]. Consistently, studies that exploited this interpretative model, like the one of Sui et al. [36], have demonstrated an improvement in neurophysiological parameters, i.e., an increase in MEPs’ amplitude and a decrease in the RMT of the damaged M1 (and changes in the opposite direction on healthy M1 when stimulated using an inhibitory protocol), and in motor and functional recovery.

However, it is crucial to note that statistically significant changes following PAS were observed only for the FMA score [36], showing a dissociation between neurophysiological and clinical measures. This phenomenon could reflect that functional changes observed in the subjects cannot be solely attributed to physiological modifications and clinical measures may be too coarse to detect these changes.

Regarding the quality of the reporting, some papers did not show their results except in the abstract or graphical form, making it complex to evaluate what was achieved [36,39]. In contrast, other studies used outcome measurement scales that are scarcely used internationally (e.g., Motor Club Assessment Scale, MCAS), making the generalization of the obtained results difficult [39]. Looking at the stroke timeframe, half of the studies evaluated patients in the subacute phase (between 1 and 6 months), three studies evaluated the chronic phase (>6 months), and one considered patients in the acute/subacute phase (<6 months). This choice may depend on the need to reconcile, on the one hand, the clinical stabilization of the patient (normally difficult to achieve in the acute phase) and, on the other, to exploit the interval of increased cortical plasticity that gradually decreases over time [54,55]. In this sense, the subacute phase seems to be the most suitable to reconcile these needs [56]. The study of Sui et al. showed significant changes in sub-acute patients following PAS [36], offering insights into applying this protocol to improve motor function even after the acute injury.

We cannot draw definitive conclusions about the number of treatment sessions due to the heterogeneity of the studies. Sui et al. found a functional improvement after 28 sessions of stimulation [36]; therefore, fewer sessions may not be sufficient to achieve this goal.

Only one study used PAS to improve lower limb function; the combined stimulation of M1 and the common peroneal nerve of the affected leg showed neurophysiological changes in the related brain areas and a functional improvement in gait [42]. The reason why the lower limb is much less investigated surely depends on its mesial area of cortical representation, less accessible with NIBS techniques. In addition, upper limb impairment most frequently afflicts stroke survivors’ daily autonomy, making the evaluation of lower limb recovery less frequent in the literature [57,58,59]. Functional improvements are observed, even in this case, in chronic patients, making the application of PAS of great interest in patients with gait impairment following stroke.

### 4.2. C/C PAS

PAS protocols aimed at improving connections between two or more brain areas (C/C PAS) have been more recently exploited to strengthen or weaken connections based on the timing of the stimulations [60]. Indeed, cortical areas are interconnected by extensive fiber bundles, both intra- and inter-hemispheric, and these reciprocal connections are crucial for the modulation of numerous activities and, in particular, motor actions [61,62]. Recent studies have shown that C/C PAS on areas involved in motor control induces significant changes, not only in neurophysiological parameters but also in motor actions [63,64], making its application of growing interest in the rehabilitation field. Consistently, several RCTs are underway to evaluate its potential in combination with various rehabilitative approaches like upper limb robot-assisted therapy [65].

Recently, several advanced PAS techniques involving combined trans-modality stimulation (e.g., between motor cortical areas and visual or acoustic ones) have been employed, remaining tied to research contexts despite promising results [66]. Therefore, although these techniques are likely to become part of stroke rehabilitation in the future, at the moment, we have limited our discussion to the study that we included in our review. Rosso et al. used a more explored “within” motor system protocol that exploited the long-range connections of the cerebellum and M1 [35], like the dentate nucleus–thalamus–cortical pathway [67]. Indeed, the contralesional cerebellum plays a significant role in the reorganization of the motor network and during the recovery process following stroke [68]. Notably, stroke patients often need to relearn basic motor strategies, a process actively governed by the cerebellum [69] that can be empowered by the simultaneous application of NIBS techniques [70].

Consistently, Rosso et al. found a significant improvement in hand function at one-month follow-up [35], leading to the hypothesis that cerebellar modulation influences motor output through morphological modifications and LTP mechanisms in the motor areas. These changes are possible in the presence of integral afferent and efferent circuits of M1 as necessary substrates for functional improvements [71]. Improvements in motor outcomes were achieved with only five days of stimulation in a group of patients who had suffered from stroke years earlier; therefore, C/C PAS seems to offer an attractive rehabilitative opportunity in chronic patients, even with a small number of sessions, probably due to its focus on the CNS, free from the influences of various peripheral factors that could reduce the effectiveness of the treatment [72].

In line with our findings, a promising role for PAS has been demonstrated in other neurological disorders, such as spinal cord injury (SCI). Neuromodulatory effects of PAS have been proposed to improve functional outcomes because of cortico-spinal and cortico-peripheral stimulation protocols [73,74]. C/P PAS, particularly, has demonstrated excellent results in terms of corticospinal transmission and functional outcome, reasonably exploiting the spike-timing-dependent plasticity principles similar to those described in this review [75,76]. However, while SCI affects areas of the nervous system that are remote from the higher brain centers, stroke damages a network of closely related and mutually influencing areas, making treatment outcomes more complex and difficult to predict [77,78]. Summarizing, although the low number of included studies makes it difficult to generalize the results, it is possible to highlight some crucial aspects. Firstly, clinical studies about PAS are highly heterogeneous in terms of stimulation protocol and parameters, stroke timeframe, session duration, number, comparators, and the motor and functional assessment scale. Moreover, PAS-empowered rehabilitation is widely used for upper limb recovery, compared to only one study using PAS for lower limb rehabilitation. Finally, the traditional C/P PAS paradigm, as described in the literature, is the most used for rehabilitative purposes, compared to the more recent C/C technique. A substantial number of studies omitted crucial information about the generalizability of the intervention, like the site and parameters of stimulation, or the specific site of the stroke lesion. Many studies evaluated the effects of PAS in terms of neurophysiological changes like MEPs and RMT, while only a few studies showed changes in clinical or functional scales used to evaluate the clinical correlation of neurophysiological aspects. Less than half of the studies reported no adverse effects or provided information to analyze their characteristics, which were in any case rare and generally minor and transient. Considering that the presence of adverse effects has not been evaluated in most of the included studies, the improvement in the reporting quality appears to be essential for a thorough analysis of the obtained results, and future work needs to adopt a methodology capable of addressing this issue.

Considering the above, it is necessary to underline that our scoping review aimed to synthesize evidence on PAS protocols in stroke rehabilitation, without considering the possible neurophysiological limitations of this technique, which, at present, require further investigation. Consequently, we were unable to quantitatively evaluate the precise interactions between PAS and rehabilitative intervention. Furthermore, it is worth noting that spinal cord circuitry gating, along with the functional status of polysynaptic descending pathways regulating the interaction between M1 and peripheral effectors, could potentially influence the outcomes of PAS protocols. However, no information is currently available on these fundamental issues. Understanding these crucial aspects could significantly impact the outcomes of PAS protocols, informing their use in neurorehabilitation.

## 5. Conclusions

The small number and the heterogeneity of the studies included in our review make it challenging to identify the role of PAS in motor rehabilitation following stroke. Despite the fact that several outcome measures have been used to quantify the efficacy of PAS in stroke rehabilitation, the study of neurophysiological modifications such as MEP and RMT is the most frequent. Our data may provide valuable information about the current use of PAS in neurorehabilitation, becoming a reference point for future studies on tailored PAS protocols identifying patients where combined stimulation can be added to motor rehabilitation to reach the best rehabilitative outcome.

## Figures and Tables

**Table 1:** Characteristics of included studies. Abbreviations: PAS = paired associative stimulation; TMS = transcranial magnetic stimulation; RCT = randomized controlled trial; RoB = risk of bias; * = conference abstract.

Unique Identifying Number	Title	Author	Year of Publication	Study Design	Country of Origin	RoB
1	Cerebello-Motor Paired Associative Stimulation and Motor Recovery in Stroke: a Randomized, Sham-Controlled, Double-Blind Pilot Trial	Rosso et al.	2022	RCT	France	6/7
2	Effect of PAS with different stimulation position on motor cortex excitability and upper limb motor function in patients with cerebral infarction	Sui et al.	2021	RCT	China	2/7
3	Five-day course of paired associative stimulation fails to improve motor function in stroke patients	Tarri et al.	2018	RCT	France	2/7
4	Effects of low-frequency repetitive transcranial magnetic stimulation and neuromuscular electrical stimulation on upper extremity motor recovery in the early period after stroke	Tosun et al.	2017	RCT	Turkey	5/7
5 *	Enhancement of cortical excitability in stroke patients after combined repetitive transcranial and peripheral magnetic stimulation	Kuznietsova et al.	2016	RCT	Ukraine	-
6 *	Study of the effects of a 5-day brain stimulation with Paired Associative Stimulation (PAS) against placebo in 28 hemiplegic patients	Tarri et al.	2015	RCT	France	-
7 *	Trial of a daily program of cerebral stimulation by TMS using a PAS paradigm in the recovery phase of stroke patients	Mohamed et al.	2013	RCT	France	-
8	Does induction of plastic change in motor cortex improve leg function after stroke?	Uy et al.	2003	Case series	Australia	3/10

**Table 2:** Clinical characteristics of the study populations, and PAS parameters and types of treatment. Abbreviations: PAS = paired associative stimulation; TMS = transcranial magnetic stimulation; PNS = peripheral nerve stimulation; ISI = Interstimulus Interval; C/C = Cortico-Cortical; C/P = Cortico-Peripheral; M1 = Primary Motor Cortex; RMT = Resting Motor Threshold; MEP = Motor Evoked Potential; ECR = Extensor Carpi Radialis; EDC = Extensor Digitorum Communis; NMES = Neuromuscular Electrical Stimulation; CS = Conditioning Stimulus; TS = Test Stimulus; FENS = Functional Electrical Stimulation; FMA-UE = Fugl-Meyer Assessment Upper Extremity; fMRI = functional Magnetic Resonance Imaging; MI = Motricity Index; BRS-UE = Brunnstrom Recovery Stages Upper Extremity; JHFT = Jebsen Hand Function Test; GS = Grip Strength; STEF = Simple Test for Evaluating and Function; BRS-H = Brunnstrom Recovery Stages Hand; MAS = Modified Ashworth Scale; MCAS = Motor Club Assessment Scale; BI = Barthel Index; M(BI) = Modified Barthel Index; MVC = Maximum voluntary contraction; ROM = Range of Motion; * = Conference abstract.

N°	Sample Size	Type of Stroke	Time from Stroke	PAS Type	Point of Application		Parameters TMS		Parameters PNS		ISI	Time of Application	Associated Treatment	Control Group Treatment	Outcome Measures
							Intensity	Frequency	Intensity	Frequency					
1	Total n = 27 Active group n = 14 (11 males, age 63 ± 14) Sham group n = 13 (10 males, age 60 ± 11)	Ischemic	Active group 202 ± 355 months Sham group 374 ± 481 months	C/C	Contralesional cerebellum (CS)	Ipsilesional M1 (TS)	CS = 90% RMT TS = 140% RMT If MEP could not be elicited: CS = 50% RMT TS = 50% RMT	0.2 Hz	-	-	2 ms	120 paired stimuli 5 sessions (1 session/day for 5 days)	Physical therapy (45 min)	Sham PAS + physical therapy (45 min)	MEP, fMRI, JHFT, GS
2	Total n = 120 Ipsilateral stimulation group n = 30 (14 males, age 44.15 ± 4.76) Contralateral stimulation group n = 30 (13 males, age 43.53 ±4.88) Bilateral stimulation group n = 30 (14 males, age 45.35 ± 5.36) Control group n = 30 (15 males, age 44.83 ± 5.18)	Ischemic	Ipsilateral stimulation group 2.0 ± 0.73 months Contralateral stimulation group 1.8 ± 0.69 months Bilateral stimulation group 1.9 ± 0.78 months Control group 1.5 ± 0.71 months	C/P	Ipsilesional stimulation group (PAS25): Ipsilesional M1 Contralesional stimulation group (PAS10): contralesional M1 Bilateral stimulation group: PAS10 (contralesional M1) followed by PAS25 (ipsilesional M1)	Ipsilesional stimulation group (PAS25): median wrist nerves innervated by ipsilesional M1 Contralesional stimulation group (PAS10): median wrist nerves innervated by contralesional M1 Bilateral stimulation group: PAS10 (contralesional median wrist nerves) followed by PAS25 (ipsilesional median wrist nerves)	120% RMT	0.05 Hz	300% of the sensory threshold	0.2 ms	Ipsilesional stimulation group (PAS25): 25 ms Contralesional stimulation group (PAS10): 10 ms Bilateral stimulation group: PAS10 followed by PAS25	90 paired stimuli 28 sessions (1 session/day for 28 days)	-	Physical therapy	MEP, RMT, FMA-UE, STEF, (M)BI
3	Total n = 24 PAS group n = 13 (9 males, age 48.6 ± 12.3) Sham group n = 11 (7 males, age 51.8 ± 12.2)	Ischemic/ hemorrhagic	PAS group 9.8 ± 5.1 weeks Sham group 10.4 ± 5.8 weeks	C/P	Lesioned M1	ECR muscle of the paretic limb	Adjusted to obtain an ECR MEP with peak-to-peak amplitude of about 1 mV	0.1 Hz	150% of the motor threshold	5 hz	25 ms	30 min PAS 5 sessions (1 session/day for 5 days)	Physical therapy (2 h)	Sham PAS + physical therapy (2 h)	MEP, FMA-UE
4	Total n = 25 TMS group n = 9 (6 males, age 57.6 ± 12.6) TMS + NMSE group n = 7 (3 males, age 56 ± 10.1) Control group n = 9 (5 males, age 61.3 ± 10.1)	Ischemic	TMS group 49.3 ± 43.6 days TMS + NMSE group 59.6 ± 58.3 days Control group 47.2 ± 41.1 days	C/P	Contralesional M1	Wrist extensors and extensor digitorum communis	90% RMT	1 Hz	Adjusted to produce the extension of wrist and fingers (90% RMT)	50 Hz	Not specified	20 min PAS 10 sessions (5 sessions/week for 2 weeks)	Physical therapy (duration not specified)	Physical therapy (duration not specified)	fMRI, FMA-UE, MI-UE, BRS-UE, BRS-H, MAS, BI
5 *	Total n = 77 (age 63.02 ± 1.21)	Ischemic	Not specified	C/P	Not specified	Not specified	Not specified	1 Hz	Not specified	Not specified	Not specified	Not specified PAS duration 10 consecutive days	Not specified	Sham PAS	MEP, RMT, MCAS
6 *	Total n = 28 (19 males, age 49.9 ± 13.5) Analyzed n = 24 PAS group n = 13 Sham group n = 11	Not specified	10.0 ± 5.1 weeks	C/P	Wrist area (Not specified side)	Wrist extensor muscle	Not specified	0.1 Hz	Not specified	Not specified	25 ms	30 min PAS 5 sessions (1 session/day for 5 days)	-	Sham TMS	MEP, FMA-UE
7 *	Total n = 18 (13 males, age 47.3 ± 12.7) PAS group n = 10 Placebo n = 8	Not specified	<6 months	C/P	Not specified	ECR	Not specified	0.1 Hz	Not specified	0.1 Hz	Not specified	30 min PAS 5 sessions (1 session/day for 5 days)	Not specified	Placebo	MEP, FMA-UE
8	Total n = 9 (6 males, age 60.6 ± 10.5)	Ischemic/ hemorrhagic	3.6 ± 10.9 years	C/P	Not specified	Common peroneal nerve in the weak limb	Intensity evoking a just-visible motor response in tibialis anterior and peroneus longus	Not specified	Intensity evoking a just-visible motor response in tibialis anterior and peroneus longus	10 Hz	35 ms	30 min PAS 1 session/day for 4 weeks	-	-	MEP, MVC, ROM, GAIT PARAMETERS

**Table 3:** Outcome measures and study results following the International Classification of Functioning (ICF) model. Abbreviations: PAS = paired associative stimulation; PT = physical therapy; TMS = transcranial magnetic stimulation; PNS = peripheral nerve stimulation; ISI = Interstimulus Interval; FMA-UE = Fugl-Meyer Assessment Upper Extremity; fMRI = functional Magnetic Resonance Imaging; FAr = Fractional anisotropy ratio; CST = Corticospinal tract; DTCT = Dentate-thalamo-cortical tracts; MI = Motricity Index; BRS-UE = Brunnstrom Recovery Stages Upper Extremity; JHFT = Jebsen Hand Function Test; GS = Grip strength; STEF = Simple Test for Evaluating and Function; BRS-H = Brunnstrom Recovery Stages Hand; MAS = Modified Ashworth Scale; MCAS = Motor Club Assessment Scale; BI = Barthel Index; M(BI) = Modified Barthel Index; MVC = Maximum Voluntary Contraction; ROM = Range of Motion; MEP-TA = Motor Evoked Potentials recorded from Tibialis Anterior muscle; MEP-PL = Motor Evoked Potentials recorded from Peroneus Longus; MVC-TA = Maximum Voluntary Contraction measured from Tibialis Anterior; MVC-PL = Maximum Voluntary Contraction measured from Peroneus Longus; MRC = Medical Research Council Scale; C/C = Cortico-Cortical; C/P = Cortico-Peripheral; M1 = Primary Motor Cortex; RMT = Resting Motor Threshold; MEP = Motor Evoked Potential; ECR = Extensor Carpi Radialis; EDC = Extensor Digitorum Communis; NMES = Neuromuscular Electrical Stimulation; CS = Conditioning Stimulus; TS = Test Stimulus; FENS = Functional Electrical Stimulation; D5 = Day five; D30 = Day thirty.

	Study	Intervention	Results			
**UPPER EXTREMITY**						
**BRAIN STRUCTURE**						
**MEP**	Rosso, 2022	Active PAS + PT vs Sham PAS + PT	*Experimental group* pre: 0.44 ± 0.62 post: 0.45 ± 0.65 fu: 0.55 ± 1.09	*Control group* pre: 0.27 ± 0.51 post: 0.33 ± 0.59 fu: 0.27 ± 0.44		*Significance* No differences within and between groups
	Sui, 2021	Ipsilateral PAS vs Contralateral PAS vs Bilateral PAS vs PT	Decrease in MEP amplitude on the contralesional side compared to before treatment. Increase in MEP amplitude on the ipsilesional side compared to before treatment. [significative differences *p* < 0.05] Decrease in MEP amplitude on the contralesional side compared to PT group. Increase in MEP amplitude on the ipsilesional side compared to PT group. [no significative differences between them] Decrease in MEP amplitude on the contralesional side and increase in MEP amplitude in the ipsilesional side in the bilateral group compared to contralesional and ipsilesional group [significative differences between them].			*Significance* Significative differences within group for the stimulation groups (*p* < 0.05) Significative differences between groups for the stimulation groups compared to PT group (*p* < 0.05) Significative differences between groups for the ipsilateral PAS25 and the contralateral PAS10 group compared to bilateral PAS group (*p* < 0.05)
	Tarri, 2018	Active PAS + PT vs Sham PAS + PT	*Experimental group* Mean (SD) surface area of MEP was 239% (230) of baseline	*Control group* Mean (SD) surface area of MEP was 154% (81) of baseline		*Significance* No differences within and between groups
	Kuznietsova, 2016	Active PAS vs Sham PAS	Reduction of latency and increase in amplitude and area in the experimental group compared to control			
	Tarri, 2015	Active PAS vs Sham PAS	No significant differences between the two groups			
	Mohamed, 2013	Active PAS vs Placebo	*Experimental group* Increase of MEP surface of 168 ± 268%	*Control group* Increase of MEP surface of 0.1 ± 48%		*Significance* No differences between groups
**RMT**	Sui, 2021	Ipsilateral PAS vs Contralateral PAS vs Bilateral PAS vs PT	Increase in RMT on the contralesional side compared to before treatment. Decrease in RMT on the ipsilesional side compared to before treatment. [significative differences *p* < 0.05] Increase in RMT on the contralesional side compared to PT group. Decrease in RMT on the ipsilesional side compared to PT group. [no significative differences between them] Increase in RMT on the contralesional side and decrease in RMT in the ipsilesional side in the bilateral group compared to contralesional and ipsilesional group [significative differences between them]			*Significance* Significative differences within group for the stimulation groups (*p* < 0.05) Significative differences between groups for the stimulation groups compared to PT group (*p* < 0.05) Significative differences between groups for the ipsilateral PAS25 and the contralateral PAS10 group compared to bilateral PAS group (*p* < 0.05)
	Kuznietsova, 2016	Active PAS vs Sham PAS	Reduction in RMT in the experimental group compared to control.			
**fMRI**	Rosso, 2022	Active PAS + PT vs Sham PAS + PT	*Experimental group* Ipsilesional M1 activity: pre: 4.3 ± 1.3 post: 3.9 ± 1.6 fu: 4.1 ± 0.8 Far_CST_: pre: 0.91 ± 0.17 post: - fu: - Far_DTCT_: pre: 0.94 ± 0.12 post: - fu: -	*Control group* Ipsilesional M1 activity: pre: 3.25 ± 1.17 post: 3.64 ± 1.45 fu: 3.62 ± 1.82 Far_CST_: pre: 0.95 ± 0.35 post: - fu: - Far_DTCT_: pre: 0.96 ± 0.15 post: - fu: -		*Significance* Not reported
	Tosun, 2017	Active TMS + PT vs Active PAS + PT vs PT	*Active TMS +* *PT group* Affected M1: Increased activation during the movements of the paretic hand in 66.7% of the group	*Active PAS + * *PT group* Affected M1: Increased activation during the movements of the paretic hand in 57.1% of the group	*PT group* Affected M1: 42.9% of the group revealed no change	*Significance* Not performed
**BODY FUNCTION**						
**Upper limb function**						
**FMA-UE**	Sui, 2021	Ipsilateral PAS vs Contralateral PAS vs Bilateral PAS vs PT	Increase in FMA-UE in stimulation groups compared to PT group			*Significance* Significative differences within group for the stimulation groups (*p* < 0.05) Significative differences between groups for the stimulation groups compared to PT group (*p* < 0.05) Significative differences between groups for the ipsilateral PAS25 and the contralateral PAS10 group compared to bilateral PAS group (*p* < 0.05)
	Tarri, 2018	Active PAS + PT vs Sham PAS + PT	No significant differences were found for time or group (*p* = 0.99). ANCOVA adjusted to the initial FMA-UE score failed to reveal a significant difference between the two groups (*p* = 0.66, 95% CI [-2.26%, 3.51%]).			
	Tosun, 2017	Active TMS + PT vs Active PAS + PT vs PT	*Active TMS + * *PT group* pre: 28.8 ± 14.9 post: 51.0 ± 11.1 *p* = 0.008	*Active PAS + * *PT group* pre: 17.3 ± 11.6 post: 30.0 ± 14.3 *p* = 0.018	*PT group* pre: 28.5 ± 18.2 post: 33.2 ± 19.9 *p* = 0.011	*Significance* Not performed between groups comparison
	Tarri, 2015	Active PAS vs Sham PAS	No significant differences between the two groups			
	Mohamed, 2013	Active PAS vs Placebo	*Experimental group* Increase of FMA-UE score: 6.1 ± 4.5	*Control group* Increase of FMA-UE score: 4.6 ± 4.1		*Significance* Not reported
**MI-UE**	Tosun, 2017	Active TMS + PT vs Active PAS + PT vs PT	*Active TMS + PT group* pre: 48.4 ± 22.8 post: 78.0 ± 17.5 *p* = 0.008	*Active PAS + PT group* pre: 28.5 ± 11.1 post: 56.8 ± 18.9 *p* = 0.018	*PT group* pre: 43.9 ± 27.0 post: 51.2 ± 27.6 *p* = 0.018	*Significance* Between-group comparison not performed
**BRS-UE**	Tosun, 2017	Active TMS + PT vs Active PAS + PT vs PT	*Active TMS + PT group* pre: 3.4 ± 1.2 post: 4.8 ± 1.1 *p* = 0.01	*Active PAS +* *PT group* pre: 2.3 ± 0.8 post: 4.0 ± 1.3 *p* = 0.016	*PT group* pre: 3.2 ± 1.5 post: 3.89 ± 1.6 *p* = 0.034	*Significance* Between-group comparison not performed
**Hand function**						
**JHFT**	Rosso, 2022	Active PAS + PT vs Sham PAS + PT	*Experimental group* pre: 5.92 ± 6.95 post: 6.00 ± 7.28 fu: 5.31 ± 6.66	*Control* *group* pre: 9.03 ± 11.7 post: 9.71 ± 10.59 fu: 10.14 ± 12.38	*Significance* Significant GROUP*TIME interaction (F (1, 26): 3.27, *p*: 0.04). There was no effect of TIME (F (2, 50): 0.6, *p*: 0.55) and GROUP (F (1, 25): 1.1, *p*: 0.29). The change in JHFT score between the active and the sham group was not significant at D5 (*p*: 0.16) but was at D30 (*p*: 0.01)	
**GS**	Rosso, 2022	Active PAS + PT vs Sham PAS + PT	*Experimental group* pre: 0.37 ± 0.27 post: 0.48 ± 0.24 fu: 0.53 ± 0.27	*Control* *group* pre: 0.37 ± 0.26 post: 0.38 ± 0.26 fu: 0.41 ± 0.29	*Significance* No effect of treatment (GROUP*TIME interaction: F (1.25): 0.60; *p*: 0.54)	
**STEF**	Sui, 2021	Ipsilateral PAS vs Contralateral PAS vs Bilateral PAS vs PT	Increase in STEF in stimulation groups compared to PT group		*Significance* Significative differences within group for the stimulation groups (*p* < 0.05) Significative differences between groups for the stimulation groups compared to PT group (*p* < 0.05) Significative differences between groups for the ipsilateral PAS25 and the contralateral PAS10 group compared to bilateral PAS group (*p* < 0.05)	
**BRS-H**	Tosun, 2017	Active TMS + PT vs Active PAS + PT vs PT	*Active TMS +* *PT group* pre: 3.3 ± 1.4 post: 4.7 ± 1.2 *p* = 0.006	*Active PAS +* *PT group* pre: 2.2 ± 0.4 post: 3.6 ± 0.9 *p* = 0.014	*PT group* pre: 3.44 ± 1.3 post: 3.89 ± 1.5 *p* = 1.02	*Significance* Not performed between groups comparison
**Muscle tone**						
**MAS**	Tosun, 2017	Active TMS + PT vs Active PAS + PT vs PT	*Active TMS +* *PT group* pre: 0.7 ± 0.9 post: 1.5 ± 1.0 *p* = 0.102	*Active PAS + * *PT group* pre: 1 ± 0.8 post: 1.0±0.5 *p* = 0.083	*PT group* pre: 0.7 ± 1.0 post: 1.0 ± 1.0 *p* = 0.180	*Significance* Not performed between groups comparison
**ADL**						
**(M)BI**	Sui, 2021	Ipsilateral PAS vs Contralateral PAS vs Bilateral PAS vs PT	Increase in MBI in stimulation groups compared to PT group			*Significance* Significative differences within group for the stimulation groups (*p* < 0.05) Significative differences between groups for the stimulation groups compared to PT group (*p* < 0.05) Significative differences between groups for the ipsilateral PAS25 and the contralateral PAS10 group compared to bilateral PAS group (*p* < 0.05)
**BI**	Tosun, 2017	Active TMS + PT vs Active PAS + PT vs PT	*Active TMS + * *PT group* pre: 66.6 ± 22.7 post: 93.3 ± 6.1 *p* = 0.008	*Active PAS + * *PT group* pre: 55.0 ± 22.1 post: 81.4 ± 20.1 *p* = 0.017	*PT group* pre: 39.4 ± 22.3 post: 50.5 ± 32.1 *p* = 0.043	*Significance* Not performed between groups comparison
**OTHER**						
**MCAS**	Kuznietsova, 2016	Active PAS vs Sham PAS	*Experimental group* Increase of MAS score: 40.4%	*Control group* Increase of MAS score: 17.1%		*Significance* Not reported
**LOWER EXTREMITY**						
**BRAIN STRUCTURE**						
**MEP**	Uy, 2003	Active PAS	MEP-TA_relaxed_ pre: 0.12 MEP-TA_relaxed_ post: 0.19 MEP-PL_relaxed_ pre: 0.8 MEP-PL_relaxed_ post: 0.8	MEP-TA_active_ pre: 0.74 MEP-TA_active_ post: 0.87 MEP-PL_active_ pre: 0.30 MEP-PL_active_ post: 0.34		*Significance* No significance for grouped data
**BODY FUNCTION**						
**Lower limb function**						
**MVC**	Uy, 2003	Active PAS	MVC-TA pre: 0.043 MVC-TA post: 0.055 MVC-PL pre: 0.014 MVC-PL post: 0.022			*Significance* No significance for grouped data
**ROM**	Uy, 2003	Active PAS	No data reported			
**ACTIVITIES**						
**Walking**						
**GAIT** **PARAMETERS**	Uy, 2003	Active PAS	No changes for 10 m timed walk, step and stride length, and cadence			

## Data Availability

No new data were created or analyzed in this study. Data sharing is not applicable to this article. All the literature used for this review is listed in the bibliography.

## Supplementary Materials

The following supporting information can be downloaded at: https://www.mdpi.com/article/10.3390/neurolint16030043/s1, Supplementary Materials: the details about the search strategy.
