# Supplementary material for: The Effectiveness of Paired Associative Stimulation on Motor Recovery after Stroke: A Scoping Review
Source: Neurol Int. 2024 May 14;16(3):567–89. doi: 10.3390/neurolint16030043 (PMC11130975; doi:10.3390/neurolint16030043)
Supplement: Supplementary file 1 [file neurolint-16-00043-s001.zip › neurolint-2938856-supplementary.pdf]

P: stroke

stroke
cerebrovascular accident*
cerebral infarction
CVA
CVAs
cerebrovascular apoplexy
apoplexy
brain vascular accident*
cerebrovascular stroke*
cerebral stroke*
acute stroke*
acute cerebrovascular accident*
hemipleg* (hemiplegia, hemiplegic)
hemipare* (hemiparesis, hemiparetic)

I: PAS

PAS
pair* associat* stim* (paired associative stimulation)
dual stim* (dual stimulation)
combined stim* (combined stimulation)
paired pulse stim*

C: -

O: motor recovery

lower extremit*
lower limb*
membrum inferius
walking
gait
locomotion
cycl*
leg
hip
knee
ankle
foot

upper extremit*
upper limb*
membrum superius
arm
shoulder
elbow
wrist
hand
grip*
motor control
motor function
motor learning
strength
long time depression
long time potentiation
cortical plasticit*
plasticit*
neuroplasticit*
synaptic plasticit*
metaplasticit*
motor cortex excitabilit*
function recover*
motor recover*
muscle strength
voluntary activation
neuromodulation
MEP
motor evoked potential
muscle recruitment
